# From the Editor's Desk

**Published:** 2017

## Introduction

This issue carries the first of what I hope will become a regular feature. On page 404, McCutcheon and colleagues publish a coronary angiogram with aggressive in-stent restenosis due to neo-intimal hyperplasia a short while after coronary stent implantation. The accompanying optical coherence tomographic images demonstrate the utility of this technology. I encourage readers to submit similar unusual or informative images.

The new oral anticoagulants remain expensive and will not be readily available in countries with limited medical resources. Warfarin will probably remain the agent of choice for some time and this almost certainly applies to many countries in Africa. Jacobs and co-workers from Tygerberg Hospital (page 346) conducted a retrospective review of patients admitted with warfarin toxicity. They identified the causes of the toxicity and the human and financial cost. An editorial by Blockman (page 344) points out that warfarin accounts for a significant number of the adverse drug reactions causing admission to hospital.

In a similar vein, Awad and colleagues (page 350) report on an investigation on medication adherence among cardiac patients in Sudan. They determined that the top four barriers to poor medication adherence among the study participants were the high cost of drugs, polypharmacy, and lack of pharmacist and physician communication with patients about their drug therapy. There is not much individual physicians can do about the high cost of drugs and polypharmacy is sometimes necessary, but we should talk to our patients to explain their illnesses and the need for adherence to medication. It is sad that this does not happen.

Balieva and co-workers report on the electrocardiographic features found in a sub-group of patients from a larger registry of patients in Africa with pulmonary hypertension, diagnosed by clinical and echocardiographic features. They compared them to ECG features found in a population known to be disease-free following extensive cardiological evaluation (page 370).

